# ERMHAN: A Context-Aware Service Platform to Support Continuous Care Networks for Home-Based Assistance

**DOI:** 10.1155/2008/867639

**Published:** 2008-08-05

**Authors:** Federica Paganelli, Emilio Spinicci, Dino Giuli

**Affiliations:** National Inter-University Consortium for Telecommunications, Department of Electronics and Telecommunications, University of Firenze via S. Marta 3, 50139 Firenze, Italy

## Abstract

Continuous care models for chronic diseases pose several technology-oriented challenges for home-based continuous care, where assistance services rely on a close collaboration among different stakeholders such as health operators, patient relatives, and social community members. Here we describe Emilia Romagna Mobile Health Assistance Network (ERMHAN) a multichannel context-aware service platform designed to support care networks in cooperating and sharing information with the goal of improving patient quality of life. In order to meet extensibility and flexibility requirements, this platform has been developed through ontology-based context-aware computing and a service oriented approach. We also provide some preliminary results of performance analysis and user survey activity.

## 1. INTRODUCTION

The growth in population ageing in most industrialized nations and the related increasing percentage of chronic disease diffusion is posing several problems at different levels of our society (political, social, and familiar/personal levels).

Statistics data provide a clear picture of the problem dimensions: according to recent results of a survey funded by the European Commission [1], Europe and Japan will experience the most pronounced ageing trends up to 2050. The share of the above 60 age group will be around 37% in Europe and even more in Japan, and slightly lower in North America (27%). The percentage of the EU population over the age of 65 is expected to reach more than 28% by 2050, with an estimated group of 80 million people that will need care and assistance services, in particular for chronic diseases [1].

New care models for chronic condition management have been proposed which define guidelines for policy planning, as well as principles for social community and health-care system organization and effort coordination. These models, usually named continuous-care models, promote home-based continuous care for chronic patients. They emphasize the fact that the effectiveness and efficiency of long-term condition care depend strongly on the capability of both the patients and their relatives to manage their cases (self-management) and on the collaboration of all involved care providers. Patients, family members, health-care teams (e.g., clinicians, general practitioner, nurses, etc.), and social community members (e.g., social workers, volunteers) should be properly informed, motivated, and prepared in order to effectively collaborate together.

One of the first examples is the Chronic Care Model (CCM), which is a conceptual, evidence-based framework developed in the USA [2]. This model proposes innovative organizational aspects aiming at improving the effectiveness and efficiency of assistance to patients affected by chronic conditions. The WHO has recently proposed the Innovative Care for Chronic Conditions (ICCC) framework, which widens the CCM in order to meet the needs of the international community [3].

Therefore, advances in information and communication technologies (ICTs) and ambient intelligence (AmI) context-aware system design are required in this framework in order to address two main objectives. The final objective is to improve the quality of life of both patients and their closest relatives. As a matter of fact, while it is obvious that chronic conditions may cause limitations to a patient's everyday activities, it should not be overlooked that they may dramatically decrease the quality of life for a patient's family members as well [4]. In order to care for the elder, relatives may be asked to make drastic life changes, such as quitting their jobs and giving up their social lives. For such a purpose, an ulterior objective is the implementation of ICT platforms capable of supporting long-term care service provision while enabling cost savings and effective heterogeneous resource management (e.g., professionals, biomedical instruments, etc.).

From the viewpoint of AmI and context-aware system designers, home-based care models pose several technology-oriented challenges. Services which should be provided by an AmI system have been classified into the following categories [5]: (a) emergency treatment (services for emergency detection and management); (b) autonomy enhancement, that is, user assistance services for primary needs and/or everyday activities (nutrition, taking medication, monitoring vital signs, etc.); comfort services, that is, services enabling better life quality (e.g., education, socialization, etc.).

These services should be adaptive, easy to use, and strongly personalized according to user requirements, needs, and disabilities. In most existing AmI context-aware systems, the focus is on the requirements of the person needing assistance (the “patient”), whereas requirements coming from the heterogeneous community of people, who are involved to different extents in patient care and assistance (the “caregivers”), are often not taken into account when designing the system.

In this paper, we describe some results obtained through our current research on the design and development of a service-oriented architecture for continuous care provisions leveraging on context-aware and mobile technologies, called Emilia Romagna Mobile Health Assistance Network (ERMHAN). Its aim is to enable the development and delivery of an extensible set of care services which allow patients to be assisted at home in a familiar environment and support the activity and mutual collaboration of care providers who are involved to a different extent in patient care and assistance. Proper and effective information sharing for action and cooperation support by involved care providers is specifically targeted, due to their work in mobility conditions, their association to different organizational domains (e.g., hospital and municipality), and their various roles (such as general practitioner, specialist, nurse, family members, etc.).

This paper is organized as follows. In Section 2, we highlight the contribution of our work and discuss existing related research activities. Section 3 describes the specific objectives of our work with special focus on the context-aware approach and in Section 4 we describe the ERMHAN system architecture and some implementation details about the two main components, the Multichannel Health-Care Service Manager and the Context Management System. In Section 5, we discuss some preliminary system evaluation results and user testing activities. Section 6 concludes the paper by highlighting the most relevant results of our work and future research directions.

## 2. RELATED WORK

Several research domains can be considered of interest in delivering AmI and pervasive health services for chronic diseases, spanning from smart homes, assistive technologies and home-based health monitoring, to context-aware hospitals.

One of the first relevant contributions has been provided by researchers working at the Georgia Tech Aware Home, a prototype of a smart home, where sensing and perception technologies are used to gain awareness of inhabitant activities and to enable services for maintaining independence and quality of life for an ageing population [6]. The INHOME project [7] aims at providing the means for improving the quality of life of elderly people at home, by developing technologies for managing their domestic environment and enhancing their autonomy and safety at home (e.g., activity monitoring, simple home environment management, flexible AV streams handling, flexible household appliance access). Among more recent works, the “ubiquitous home” is a real-life test-bed for home-based context-aware service experiments [8] in Japan. A set of implemented context-aware services has been evaluated by means of real-life experiments with elderly people.

In the field of assistive technologies and home-based health monitoring systems, several examples exist: Vivago is an alarm system which provides long-term user activity monitoring and alarm notification [9]; the CareMedia system [10] uses multimedia information to track user activities.

The “hospital of the future” prototype [11] is an example of a context-aware computing system in a hospital environment. It consists of a series of context-aware tools: an electronic patient record (EPR), a pill container, and a hospital bed which displays relevant patient record information, such as the medicine schema, according to contextual information (e.g., nurse position, patient, medicine tray). Muñoz et al. [12] have recently proposed a context-aware mobile system where mobile devices are capable of recognizing the setting in which hospital workers perform their tasks, and let users send messages and access hospital services according to these contextual elements.

Despite the multitude of relevant contributions in the above-mentioned research fields, only recently research activities on pervasive services for ageing and chronic disease management have begun addressing these requirements by means of a holistic approach, taking systematically into account standard guidelines and reference models for continuous care. Consolvo et al. [4] have applied social network analysis methodology to the study of continuous care networks; they conducted a series of interviews in order to explore the space of eldercare (i.e., who was involved in the care, what types of care were needed, and what types of care were being provided); based on user study results they offer some design guidelines for the development of successful computer-supported coordinated care (CSCC) systems.

Pervasive self care is a conceptual framework for the development of pervasive self-care services [13]. This study has been promoted in the framework of self care, an initiative by the Department of Health in the UK that aims at treating patients with long-term conditions near home. The proposed reference model, inspired by the principles of service-oriented architecture (SOA), distinguishes three main spheres: the body sphere (a body area network supported by a router which interacts with body sensors and with the home sphere); the home sphere (a home server that collects and preprocesses sensed data), and the self-care service sphere (the data processing and sharing subsystem).

Some experimentation results are given in [14], where a telemedicine system is used for the home care of patients suffering from chronic obtrusive pulmonary disease (COPD); the integrated telemedicine system provides professionals with shared and ubiquitous access to patient health records and patients with direct access to nurse case manager, telemonitoring, and televisit services.

### 2.1. Contribution of our work

The design of ICT tools for chronic disease management should take into account flexibility and extensibility requirements. These requirements are common to all kinds of distributed systems, but especially are applied to this application domain, due to its intrinsic characteristics, such as different national and local regulation frameworks, the heterogeneity of health centers and communities involved in care service delivery, and the patient's health status development over time. In such a complex and changing environment, cost-effective solutions should be conceived as extensible and flexible service platforms. For that reason, while the research activities surveyed above have focused on the conceptual design or implementation of applications that target specific chronic diseases and not on a specific chronic disease from the very beginning, our approach has been to design and deploy a service platform that provides general purpose services and could be easily extended and specialized in order to match specific requirements of real cases.

The aim of our research has been to design and implement ERMHAN, an extensible service platform supporting care teams in providing long-term assistance services. Extensibility and flexibility of the ERMHAN service platform are mainly achieved by means of modular and service-oriented design and the adoption of open and standardized data formats and communication protocols.

The platform design is based on the definition of basic functional blocks and their interconnection by means of web services standards. As a matter of fact, web services are recognized as an open and standardized way of achieving interoperation between different software applications, running on heterogeneous platforms and/or frameworks [15].

Semantic web technologies have been applied to data representation and processing in order to provide instruments that ease the development of pervasive and personalized care services. More specifically, semantic web is used in order to (a) represent knowledge by means of ontology-based formalisms; (b) reason over knowledge using rule-based and ontology-based engines; (c) apply reasoning techniques in order to implement personalized healthcare plans.

The prototype we have developed provides basic and general-purpose services for information sharing, distributed, and multichannel personalized access to patients' records and personalized real-time monitoring and alarm management on a per patient basis. Implemented services include access to complete and updated patient records (including patient health status, description of care provider interventions), even in mobility conditions (at the hospital or at patient's home) and through different devices (at least personal digital assistants and desktop PCs); notification of patient health status conditions and alarms, but without overwhelming care operators with too much information (this might have the drawback of disturbing users and providing them with “no information”). More effective information delivery could be achieved by routing intervention requests according to patient health status gravity and required expertise for intervention.

Based on its flexibility, the ERMHAN service platform can be specialized to address the needs of specific patient cases. To achieve this objective, the basic services provided by the ERMHAN platform can be integrated with those offered by other systems, such as assistive technologies, home automation systems, specific chronic disease prognoses, and diagnosis systems [16]. In the following sections, we provide further details about the modeling approach and system architecture of ERMHAN platform.

## 3. CONTEXT-AWARE MODELING AND REASONING FOR HOME-BASED CARE

The objective of this research is the design and prototypal development of a context-aware mobile service platform (ERMHAN) for long-term home-based care services delivery to chronic patients.

ERMHAN aims at supporting the main characteristics of emerging chronic care models: (a) home-centered long-term patient followup; (b) shared care provided by a network of heterogeneous care providers (named “continuous care network”); (c) adaptation of care plans on a per patient basis.

With the term “continuous care network” we refer to the broad range of people involved to different extents in the care of the elder, such as patient relatives, the multidisciplinary teams at different health-care levels (e.g., general practitioners, specialists), social operators, volunteers, and so forth. They belong to different organizations (e.g., hospitals, municipality, private institutions), may work in more than a single location, and do not usually have regular face-to-face contacts with other network members.

Due to the complexity of the care network, its ad hoc nature, and loosely coupled structure, we have identified a set of basic services to be provided to care providers: ubiquitous access to patient-health records (via mobile device or desktop PC); role-based information sharing among different network members belonging to different organizations; capability of adapting health plans on a per-patient basis. More specifically, health-plan personalization is implemented in ERMHAN in terms of personalized context-aware rules for alarm detection and management, as described in the next paragraph.

Even if the aim of our work is to provide services to care providers, and consequently developing assistive technologies are not the main target of this work, ERMHAN provides some basic services for patient assistance, for example, medication reminders, manual alarm activation, and communication request services. As the focus of this work is on the architecture for service integration and delivery, human computer interaction (HCI) issues, such as usability and accessibility, have not been taken into account at this stage of our study.

**Figure 1:**
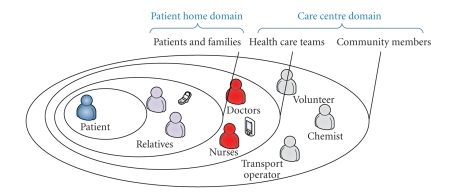
ERMHAN applications scenario.

The ERMHAN reference application scenario consists of two main domains (see Figure 1):

- the patient home, that is, the environment where the patient lives, often with some relatives, and where patient monitoring and assistive technologies would be deployed;
- the care centre, that is, the domain of the organization responsible for care service integration and delivery (e.g., hospitals, nursing homes or municipalities). For the sake of simplicity, in our scenario this is presented as a single logical point of care, providing ICT services to care operators who can belong to different organizations. Actors considered in our scenario are doctors (general practitioners and specialists) and nurses. More complex scenarios involving other organization domains (e.g., voluntary associations, pharmacies, etc.) will be analyzed in future research activities.

### 3.1. Context awareness

This paragraph describes what we do intend for “context awareness” in the framework of home-based continuous care and the context model that has been adopted in ERMHAN.

Context awareness is a general concept that refers to the capability of a system to be aware of its physical and logical environment and to intelligently react according to this awareness. The definition of context is likely to change according to the application domain and related purposes [17].

In our application scenario, context awareness is meant as the capability of the system to acquire and interpret relevant information with respect to the main goal (patient quality of life) and to perform actions aiming at achieving such a goal. Thus, the context may include information about patient health status, inferred by sensed biomedical parameters (e.g., vital signs), and home environment (e.g., temperature and relative humidity). Even social context (i.e., information regarding people populating the patient care network) may be considered as relevant information to be exploited for context-aware assistance service delivery. The acquisition of these context data can be profitably exploited by the system in order to infer relevant situations related to patient status, (such as critical condition and alarm detection) and trigger proper actions in order to facilitate the care providers' operations (i.e., alerting care providers for intervention).

#### 3.1.1. ERMHAN context model

An ontology-based context model, written in Web Ontology Language (OWL [18]), is employed throughout the entire process of sensing, interpreting, managing, and exchanging context information. We have extended a general purpose ontology-based context model [19] with concepts and relations describing the home care setting.

The OWL-based context model is used in order to represent system knowledge about patient health status by means of ontology-based formalisms. The context model provides a uniform representation of data coming from heterogeneous sources, such as biomedical sensors, home monitoring systems and users' manual input. Ontology and rule-based reasoning are applied over the context model in order to check the consistency of context information and to infer further knowledge. Rule-based reasoning is applied in the ERMHAN system in order to infer patient health conditions, detect alarm conditions originating from a patient health status or other events (e.g., the patient does not take medication), and select the alerting policy which is most suitable according to alarm level and other context information (e.g., availability of care providers for intervention).

The main advantage of this approach consists in representing the business logic of the system in an explicit and declarative way. Thus, adaptation of health plans on a per patient basis can be realized by allowing care providers to specify rules tailored to the patient case.

Hereafter we describe the main features of the context modeling and reasoning capabilities implemented in the ERMHAN system. A complete description of our ontology-based context model for home health monitoring and alerting in patient care networks is out of the scope of this article and is fully described in [20].

The context model is composed of the following parts.

1. The *patient-domain ontology* includes context items about patient biomedical parameters, location, and activity. This information is used by the system to automatically infer patient health status and detect alarm situations by means of rule-based reasoning. Biomedical parameters instances are represented together with some relevant properties, such as measurement values and parameter ranges. Each range is specified in terms of upper and lower thresholds and related alarm level; when a measured value falls out of the thresholds, an alarm of the corresponding level is detected. Patient health status is thus determined by comparing biomedical parameters' measured values with a set of parameter ranges. We have specified four basic alarm levels (very low, low, medium, high), but the model can be easily extended to include further levels.
2. The *home environment ontology* includes context data that describes the patient home environment. For instance, monitoring environmental parameters (such as temperature and relative humidity) are needed in order to maintain a healthy environment and detect alarm situations. Alarm triggering based on environment parameters is analogous to the one based on biomedical parameters, described above.
3. The *social context ontology* represents care network resources (health teams, social community members, etc.) and their relations. Care network members that are represented as concepts in this ontology include the patient, patient relatives, who take active part in implementing the assistance plan, health operators (medical and paramedical staff) who are in charge of patient care; social community organizations which offer assistance services (e.g., transport and companion services). The social context ontology is also used to represent other information which is considered relevant to the ERMHAN application purposes, such as the availability of a health operator for an intervention and the distance of a relative from patient location (travel time). At present, users can provide this information via a web form provided by the profile management services, by choosing among predefined values (e.g., “available,” “busy,” “not available”). As part of future work, we will investigate the use of context-aware systems that can help in semi-automatically updating and managing such information (e.g., GPS positioning and inference mechanisms).
4. The *alarm management ontology* represents the policy that should be instantiated to manage an alarm. Such policy describes the steps that should be performed in the event of an incoming alarm. Rule-based reasoning over this ontology model is used to select the suitable alarm handling policy according to different context items, such as the incoming alarm level, the patient identifier, and the availability of care team members (i.e., the information represented by the social context ontology).

#### 3.1.2. Rule-based reasoning examples

As mentioned above, the ERMHAN system uses rule-based reasoning over the context ontology instances for triggering alarms and defining policies for handling alarms.

Alarm triggering is based on the analysis of context data represented in the Patient and home-domain ontologies. We have defined a set of first-order rules so as to determine if an alarm has to be triggered and which level should be activated according to measured context data and corresponding thresholds. For instance, the following rule triggers an alarm classified as “high” when the heart rate frequency is less than 40 beat/minute and the systolic blood pressure is higher than 160 mm/Hg:

IF (HeartRateFrequency<40 AND SystolicBloodPressure>160) THE HealthStatus has AlarmLevel “HIGH.”

Likewise, when an alarm has been triggered, rule-based reasoning is used to determine which alert policy should be actuated for handling the alarm situation. In ERMHAN, an alert policy is represented as a set of instructions specifying whom should be alerted and how (via SMS, mail, phone call) and if acknowledgment is required. In Table 1, we define a basic example policy for each alarm level.

For instance, we suppose that when a “MEDIUM” level alarm is detected, the system should perform the following actions (see Table 1).

- Alerting an operator via SMS or email. First, a general practitioner is alerted. If he/she does not send the acknowledgment within a fixed-time interval, other operators (e.g., nurses) are alerted until one of them sends an acknowledgment. At the end, if no acknowledgment is received, the system alerts an emergency operator, available 24/24 and 7/7. The list of operators to be contacted is dynamically created by the system by selecting the caregivers which are assigned to that patient and are “available” for intervention.
- At the same time at least one of family members should be alerted, but acknowledge is not required.

Adaptation of system behavior on a per-patient basis can thus be achieved by care providers by specifying personalized alarm levels, parameter thresholds, and alert policies tuned to the individual's conditions.

## 4. ERMHAN SYSTEM ARCHITECTURE

**Figure 2:**
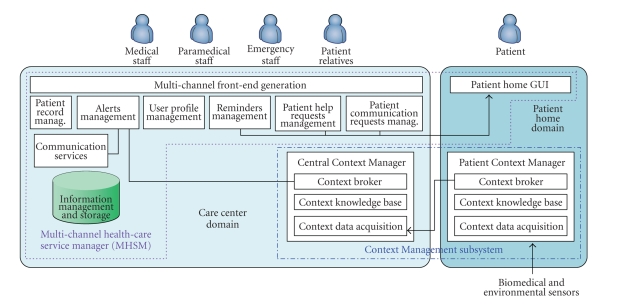
ERMHAN architecture.

The ERMHAN system architecture, shown in Figure 2, is made of the following main components.

The Multichannel Health-Care Service Manager (MHSM). This component delivers mobile health-care context-aware services to patients, relatives, and care providers, targeting a variety of user devices. It is deployed in the care centre domain and includes a front-end component deployed in the patient-home domain.

The Context Management system. This component deals with the acquisition of context data from heterogeneous sources, the management, and storage of ontology-based context instances, reasoning over context knowledge, and the delivery of relevant contextual knowledge to context-aware applications (e.g., MHSM).

MHSM and Context Management systems have been designed as completely separate and independent modules. This has been done in order to strongly decouple context data acquisition, management, and context-aware service delivery. Communication among these components is based on web service interfaces.

### 4.1. Multichannel health-care service manager

The Multichannel Health-Care Service Manager (MHSM) is the ERMHAN component that provides mobile context-aware health-care services to end-users [21]. MHSM provides end-users with proper services according to their roles and related requirements. ERMHAN services are specifically targeted to care-provider requirements. Thus, end-user roles which have been taken into account are medical, paramedical, and the emergency centre staff. Nonetheless, we have also considered some basic requirements for patients and patients' relatives.

**Medical staff**

The medical staff must be constantly informed about the patient's conditions. Each member can access ERMHAN services remotely, via a desktop PC or a PDA. Available services include the following.

(•) Patient record management. Medical staff members can access and update the patient record using a personal digital assistant (PDA) or desktop PC. Information sharing among care professionals is achieved by providing them with the capability of accessing a uniform view of patient information. Permission for reading and modifying patient record fields is tailored according to the user's role (e.g., general practitioner, specialists, etc.)
(•) Alerts management service. When an alarm is triggered (manually by the patient or automatically by the Context Management system), the alerts management service contacts the staff through the appropriate channels (e.g., mail and/or SMS). As specified by the alert policy, alerts can be sent as informative messages or as intervention requests, depending on the alarm gravity level. In the latter case, the caregiver is asked to acknowledge the alert reception and to confirm the intervention request. More details about the alert policy are provided in the next subsection.
(•) Profile management service. The medical staff members can specify their own availability for the intervention (e.g., available, not available, busy). This information is taken into account by the rule-based reasoning process used to select the operators which should be contacted in the alert policy.

**Paramedical staff**

The paramedical staff (e.g., general assistance operators, nurses, etc.) can access the system remotely, by means of a PC or a PDA. Similarly to the medical staff, but with different role-based access rights, they can access the following services: patient record management, alerts management services, and profile management services.

**Emergency staff**

The emergency staff is composed of “always available” operators. They are notified by the alerts management service in order to respond to emergency situations (highest critical alarm level) or when other health operators have not responded to lower level alarms (e.g., medical staff).

**Patient**

We considered the following patient requirements for eliciting a basic set of services: the capability of easily communicating with relatives or health operators, manually activating alarms, and having support for implementing the health assistance plan. Requirements depending on specific patient conditions (e.g., physical impairments, cognitive disabilities, etc.) have not been considered in this study. The patient is equipped at home with a Tablet PC and uses services through a Front-End available on the Tablet PC touch screen. More specifically, services which are available to patients at home include the following.

(•) Reminders service: the system alerts the patient that medication has to be taken. The alert signal is an audio and video alarm which is remotely triggered on the front-end deployed at patient home.
(•) Help requests: the patient can manually generate an alarm, in order to request an urgent intervention by the emergency staff.
(•) Communication requests: the patient can request to be contacted by some relatives.

**Patient relatives**

The patient's family members provide general assistance to the patient during daily routine activities at home and should be kept informed about patient conditions when outside the home (via the alert management service and profile management service).

Services targeting relatives can be accessed via mobile devices with minimal technical requirements (i.e., cell phones with an XHTML browser).

**Multichannel health-care service manager implementation**

The MHSM has been designed and developed as a multichannel web application written in Java language, leveraging the J2EE JSP/Servlet technology framework. The MHSM has been developed as a 3-tier architecture including the following.

(1) A back-end tier, named MHSM information management and storage, handles and stores the following information resources: the patient record, containing patient personal data, personal assistance plan, vital sign thresholds, medical, sanitary and assistance diaries, and prescriptions (e.g., taking medication, vital sign measurement scheduling); user profiles, containing end-user personal data, organizational roles, caregiver availability status (for medical and paramedical staff and patient relatives); organization models, representing the care network structure, (i.e., care network members and patients under care).

(2) An intermediate tier hosts the business logic of the ERMHAN Services (patient record management, alerts management, profile management, reminder management, patient help, and communications request management).

(3) An upper tier, composed of a multichannel front-end generation service, generates user interfaces tailored to the capabilities of the different end-user mobile devices (e.g., display size, resolution, memory and processing capabilities, etc.). The target devices for this scenario are PDAs, Tablet PCs, cell-phones, and laptops. The model view controller design paradigm [22] has been adopted in order to properly manage user interface generation, business and navigation logic, and content generation.

The platform uses the web services technology standards (SOAP, WSDL) to interact with external components, such as the Context Management system. The underlying DBMS is MySQL. A communication management service implements an SMS gateway and the SMTP protocol in order to provide SMS and e-mail alerts to care network members (the adoption of further communication channels and protocols will be evaluated in the future). This component has been designed and developed by the industrial partner of the KAMER Project (HP Italy).

### 4.2. Context Management system

This section describes the Context Management (CM) system architecture that we have developed to ease the implementation of context-aware assistance services for chronic patients.

The CM system is based on a general-purpose framework providing basic features for context data acquisition, reasoning, and delivery [19]. It is composed of two different node types.

- The Patient Context Manager (PatientCM). This node is deployed at the patient site. It acquires data retrieved by biomedical and environmental sensor networks. These data are preprocessed by the PatientCM in order to detect abnormal conditions and are transmitted to the MHSM in order to update patient records with new measurement values. Sensed data are injected into the patient and home-domain ontology instances and rule-based reasoning processes are performed over this context base in order to detect incoming emergency situations and trigger corresponding alarms. The Central Context Manager is then notified of the occurrence of an alarm.
- The Central Context Manager (CentralCM). This component is deployed in the care centre domain. When an alarm situation has been detected by a PatientCM, the CentralCM processes context information about patient health status and care team member availability, in order to define an alert policy for informing care team members and request their intervention in case of emergency. It handles the alarm management and social context ontologies by creating related instances updated with context information about patient health status, triggered alarm levels and information about care team member availability. Based on this context information, reasoning rules are applied in order to define the proper alert policy for alarm handling. The resulting alert policy is then sent to the MHSM for its implementation.

Both the Patient and the Central Context Managers include the following main blocks.

Context data acquisition: this component receives context data from context providers via SOAP messages (e.g., web service-enabled sensor networks), or via sensor adapters.

Context knowledge base: this is a knowledge base composed of ontology-based context models and instances stored in a database, a rule-based reasoner and rule files. This component is based on Jena, an open source Semantic Web framework [23].

Context broker: this component distributes context data to external components by notifying the interested applications when the context has changed or by providing updated information in a request/response chain. Both notification and request/response paradigms are implemented through a SOAP message exchange [24].

In each Context Manager, the communication between internal components has been implemented according to the observer design pattern, that is, through suitable EventListeners that listen for events and encapsulate the information to be processed [25]. Therefore, such decomposition allows each internal component to process its own specific information independently.

The CentralCM and PatientCM have been implemented as J2EE web applications and their external interfaces are exposed as web services.

#### 4.2.1. Alarm detection and management scenario

**Figure 3:**
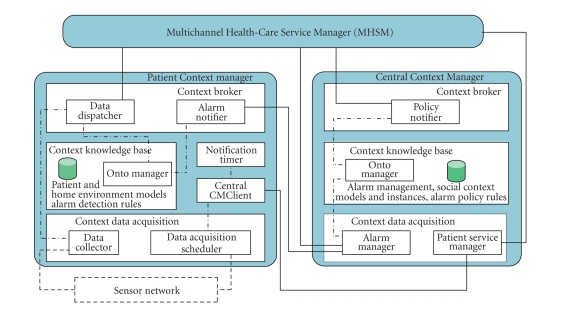
ERMHAN Context Management system.

**Figure 4:**
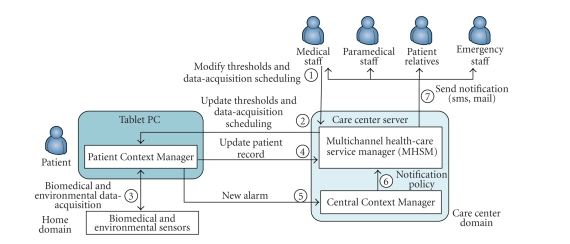
Alarm detection and management scenario.

This paragraph provides further insight into the Context Management system architecture (see Figure 3) by describing the system behavior in an alarm detection and management scenario. Figure 4 provides a simplified graphical representation of this scenario as a sequence of numbered steps.

General practitioners and nurses can access and modify patient health information through the MHSM interface (Step 1). In particular, general practitioners can update remote monitoring parameters, such as scheduling sensed data acquisition and related alarm thresholds. For instance, a practitioner can specify that the body temperature should be measured at 7 am and 5 pm every day, that a measured value higher than 38°C should trigger a MEDIUM-level alarm if reached at 7 am, and a high-level alarm if reached at 5 pm. The MHSM sends this information to the CentralCM which routes the message to the proper PatientCM (Step 2).

The *CentralCM.PatientServiceManager* component offers a web service interface which can be invoked by the MHSM in order to communicate messages such as updates of patient diagnostic test scheduling and vital sign alarm thresholds. The CentralCM manages a message queue for each PatientCM and messages that come from the MHSM are then routed to the proper message queue. The PatientCM.CentralCMClient component periodically polls the *CentralCM.PatientServiceManager* to request new messages.

This periodical polling is also used to monitor the status of both the PatientCM operation and the connection between the PatientCM and the CentralCM. When the CentralCM does not receive the expected periodical poll by a PatientCM (this condition may be caused by connection failures or PatientCM malfunctions), it triggers a corresponding alarm and the emergency staff is alerted to handle this technical problem by activating the required procedures.

The *PatientCM.CentralCMClient* parses the message payload and produces suitable events, such as TimingEvents for scheduling biomedical data acquisition.

New timing events are consumed by the *PatientCM.DataAcquisitionScheduler*. According to the prescriptions contained in the TimingEvents objects, the scheduler allocates corresponding timers triggering the patient's biomedical data acquisition (e.g., body temperature, heart rate frequency) from web service-enabled body sensor networks (Step 3).

The *PatientCM.DataCollector* exposes a web service interface which is invoked by the sensor networks to communicate new acquired vital sign values. These data are then passed on to the DataDispatcher, that notifies the MHSM of these data for patient record updating (Step 4) and the OntoManager module for updating the Patient and home environment ontologies instances. Rule based reasoning is then applied to the knowledge base in order to detect possible alarms and produce AlarmEvents. For instance, if the sensed body temperature is higher than 38°C a MEDIUM-level alarm is triggered. The *PatientCM.AlarmNotifier* notifies a new AlarmEvent to the CentralCM, by invoking the *CentralCM.AlarmManager* component (Step 5).

The *CentralCM.AlarmManager* receives new incoming alarms notified by the PatientCM and routes them to the *CentralCM.OntoManager* which has to create an alert policy for alarm handling. Firstly, it feeds the knowledge base (composed of the alarm management and social context ontologies) with instance data, such as attributes of the AlarmEvent (patient identifier and alarm level) and updated information about the patient social context (health operators availability) which are made available by the MHSM. Then, it applies rule-based reasoning to produce the alert policy.

The *CentralCM.PolicyNotifier* is the component which sends the alert policy to the MHSM, by invoking a specific web service (Step 6). The MHSM then implements the policy by sending proper alerts to the contact lists (Step 7).

Filtering techniques are implemented by the *CentralCM.AlarmManager* to handle sequences of alerts related to the same emergency situation. As a matter of fact, while an alarm is being handled, notification related to the same situation can be generated again by the PatientCM (e.g., because of new vital sign measurements). The filtering technique is thus applied to avoid overwhelming health operators with redundant alerts.

## 5. SYSTEM EVALUATION AND TESTING ACTIVITIES

This section discusses the ERMHAN system results in terms of performance evaluation and user testing activities.

### 5.1. Context Management system performance evaluation

The following considerations are concerned with a performance estimation of the ERMHAN Context Management system obtained by collecting average alarm generation and transmission times in a basic configuration. This configuration is composed of one PatientCM and one CentralCM. The PatientCM is geographically located 40 Km away from the CentralCM. The server hosting the CentralCM has an external IP and accesses the Internet through the university wide area network. The PatientCM can access the Internet through a standard low-band with DSL connection (640 Kbps). More precisely, the PC hosting the PatientCM has a wireless connection to an 802.11g DSL gateway. In particular, we have considered the following hardware configuration.

1. PatientCM: AMD Athlon64 3200+, 1024 Mb RAM.
2. CentralCM: Intel Pentium 4 2.0 GHz, 768 Mb RAM.

The performance measurement is based on the following parameters: the alarm detection time (**T**_**ALARM**_) which is the time interval between the acquisition of an out-of-range biomedical parameter value and the triggering of the corresponding alarm obtained by means of rule-based reasoning (Section 3.1.2); the transmission delay (**T**_**TRANSM**_) which is the time needed for transmitting a message through the connection between PatientCM and CentralCM; the alert policy time (**T**_**POLICY**_), which is the time elapsing at the CentralCM side between the reception of an incoming alarm originating from the PatientCM and the generation of the corresponding alert policy. The sum of these time intervals determines the overall time needed for the alert policy generation from time the out-of-range biomedical parameter is acquired (**T**_**MANAGEMENT**_):

(1) $$T_{\text{MANAGEMENT}}\;=\;T_{\text{ALARM}}\;+\;T_{\text{TRANSM}}\;+\;T_{\text{POLICY}}.$$

**Figure 5:**
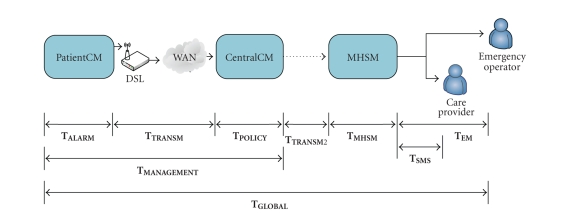
Time intervals for ERMHAN performance evaluation.

The value of **T**_**MANAGEMENT**_ does not vary significantly with respect to alarm levels. Nonetheless, alarm levels influence the kind of alert policy generated by the CentralCM and implemented by the MHSM. An evaluation including also policy implementation and the notification to at least one care provider should also take into account the following time intervals (see Figure 5): the time needed for transmitting the alert policy from the CentralCM to the MHSM (**T**_**TRANSM2**_); the time needed by the MHSM for instantiating the alert policy (**T**_**MHSM**_); the SMS latency (**T**_**SMS**_) which is the delay for transmitting SMSs to health operators; the delay for transmitting alert to the care centre in order to notify emergency operators (**T**_**EM**_).

The formula for calculating the global time interval (**T**_**GLOBAL**_) from the alarm detection to the alert notification to care providers differs according to the alarm level and corresponding alert policy.

For VERY-LOW- and LOW-level alarms, the following formula is applied:

(2) $$T_{\text{GLOBAL}}\;=\;T_{\text{MANAGEMENT}}\;+\;T_{\text{TRANSM}2}\;+\;T_{\text{MHSM}}\;+\;T_{\text{SMS}}.$$

For MEDIUM-level alarms, the calculation has to take into account the fact that health operators (general practitioners and nurses) are notified in sequence: a first operator is alerted via SMS, if he/she does not respond within a specified time interval (**T**_**TIMEOUT**_), another operator is notified. After **N** failed attempts (**N** is specified in the alert policy), the alarm is communicated to an emergency operator (**T**_**EM**_).

If a care provider confirms the SMS reception after *n* attempts (*n* ≤ *N*),

(3) $$\begin{array}{ll} T_{\text{GLOBAL}} & =\;T_{\text{MANAGEMENT}}\;+\;T_{\text{TRANSM}2}\;+\;T_{\text{MHSM}}\\ & \;+\;n(T_{\text{SMS}}\;+\;T_{\text{TIMEOUT}}) \end{array}$$

otherwise

(4) $$\begin{array}{ll} T_{\text{GLOBAL}} & =\;T_{\text{MANAGEMENT}}\;+\;T_{\text{TRANSM}2}\;+\;T_{\text{MHSM}}\\ & \;+\;N(T_{\text{SMS}}\;+\;T_{\text{TIMEOUT}})\;+\;T_{\text{EM}}. \end{array}$$

For HIGH-level alarms the following formula is applied:

(5) $$T_{\text{GLOBAL}}\;=\;T_{\text{MANAGEMENT}}\;+\;T_{\text{TRANSM}2}\;+\;T_{\text{MHSM}}\;+\;T_{\text{EM}}.$$

As mentioned above, this paragraph focuses on performance estimation of the Context Management system in delivering the most critical service (alarm detection and handling). Thus, our evaluation is based on measured values of *T*_ALARM_, *T*_TRANSM_, and *T*_POLICY_. Values of further parameters (**T**_**TRANSM2**_ **T**_**MHSM**_,**T**_**SMS**_ and **T**_**EM**_) are not available for dissemination as they have been collected by the industrial partner (HP Italy). Moreover, values of **T**_**SMS**_ and **T**_**EM**_ are dependent on the characteristics of the network infrastructure. In a real implementation scenario, these parameters might be optimized according to appropriate technological choices, such as using public or professional radio-mobile networks for SMS delivery (i.e., GSM and TETRA, resp.) and a dedicated fixed line for communicating with the emergency operators, as well as establishing proper service level agreements with network operators.

To estimate the performance of the implemented system in detecting and managing alarms, we have simulated the generation of 60 sample alarms for each of the four alarm levels (“Very low,” “Low,” “Medium,” and “High”). Such samples have been acquired by monitoring the activity of the Context Management system upon an interval of five working days, during working hours, in order to stress the system during worst traffic conditions (i.e., peak traffic hours). In more detail, experiments were performed during two time slots: 10:00 A.M.–2:00 P.M. (*Time Slot 1*) and 2:00 P.M.–6:00 P.M. (*Time Slot 2*).

Table 2 reports the mean and standard deviation values in milliseconds of alarm detection time (**T**_**ALARM**_), alert policy time (**T**_**POLICY**_), and the overall alarm management time (**T**_**MANAGEMENT**_), which is calculated as the sum of **T**_**ALARM**_, **T**_**POLICY**_, and the transmission delay (**T**_**TRANSM**_).

Measured values of **T**_**ALARM**_ as well as **T**_**POLICY**_ do not vary significantly across alarm level. **T**_**POLICY**_ measured values are higher than **T**_**ALARM**_ ones as the major part of the processing load is leaning to the PatientCM side, according to the architecture of the Context Managers.

Measured values of the overall **T**_**MANAGEMENT**_ vary significantly according to network traffic conditions which directly influence **T**_**TRANSM**_ values. In this experiment, we can estimate an overall alarm time swinging between 8 and 10 seconds, with a transmission delay that can be evaluated in about 5 seconds. The higher values of the standard deviation for alarm times can be charged on interferences in the PatientCM wireless connection and on the network conditions in the restricted observation window (five working days) considered for this evaluation.

We expect that a more performant connection would significantly improve the Alarm Management Time, and a wider observation interval (i.e., one month) would produce more uniform statistics for this indicator.

In order to minimize reasoning tasks execution time (inference on ontology instances), we perform reasoning on small ontologies populated by a small number of instances, since ontological reasoning execution times grow at least linearly with respect to the ontology size [26]. This time could be further optimized by performing part of the reasoning task before the service request. Future research activities will thus focus on analyzing which reasoning tasks could be performed prior to service requests and consequently on reengineering the PatientCM and CentralCM.

### 5.2 User trials

As ERMHAN services have been designed to support care networks and thus to address requirements of care providers, testing is primarily to be focused on evaluating health operators' acceptance of implemented features.

A trial has been performed in a nursing home in Piacenza for system evaluation by professional caregivers. Further trials are planned in a nursing home in Florence in the near future, for more extensive evaluations by chronic condition patients. For these future tests, a sensing system for vital signs and environmental monitoring will also be deployed. Testing activities will thus focus also on evaluating patient acceptance with regard to deployed services (i.e., monitoring, medicine reminder, help, and communication request services).

In the testing stage already conducted in Piacenza, biomedical and environmental sensing was simulated by a web application. Biomedical parameters that were represented in the model included heart rate frequency, pulse oxymetry, systolic and diastolic blood pressure, body temperature, and glycemia. The web application provides services for manual input or predefined scenario simulation for context data acquisition.

During each session, practitioners were equipped with mobile devices and PCs. The project staff took care of simulating the acquisition of biomedical and environmental parameter values and the occurrence of alarms through the dedicated web interface; health operators were asked to react to such events by using the ERMHAN system as they were in the “real world,” such as ignoring alerts or confirming the intervention request reception and taking charge of the case. Practitioners were also asked to perform day-by-day operations through the system, such as accessing and updating patient records, modifying the scheduling of biomedical parameters acquisition and related alarm thresholds. At the end of these trials, the testers' opinions about system features have been collected through interviews and questionnaires.

This first trial session was conducted with 11 test users (including both general practitioners and nurses). As a consequence, we are far from having statistically significant data available, but we are able to illustrate some preliminary results which can be drawn from questionnaire responses. A large majority of users were quite satisfied by the system's overall features (more than 60%). The capability of accessing patient health records has been judged useful and easy to use (but some users had already tested similar features in other experimental systems). The alarm management service and the capability of specifying availability for intervention were judged especially useful (42% of users) and useful (50% of users). Most users appreciated the capability of remotely modifying the alarm thresholds and data acquisition scheduling on a per-patient basis (18% expressed high appreciation, 73% good appreciation, 9% were neutral). A group of users (30%) complained about some misalignment between MHSM patient record presentation and paper-based records in use in their nursing home (especially in terms of use, information organization and classification). This aspect will be further investigated in future testing activities and properly analyzed when defining a methodology for customized deployment if ERMHAN is applied industrially.

## 6. CONCLUSIONS

In this paper, we have presented ERMHAN, a context-aware mobile service platform supporting mobile caregivers in their daily activities. ERMHAN has demonstrated its capability of providing an extensible set of services aiming at supporting care networks in cooperating and sharing information for the goal of improving a chronic patient's quality of life.

ERMHAN has been designed as a modular system, and its components have been implemented by adopting standard technologies (e.g., Internet protocols, XML, Web services). This approach makes the system easily extensible to match specific patient requirements within an ambient intelligence environment.

Future work will concentrate on security and dependability issues as well as on extending the features provided by the service platform. In the home domain, this will mainly consist in deploying body and home sensor networks, and integrating input/output devices for patients (e.g., alarm button and TV displays). As for the care centre domain, the main developments will include designing graphical user interfaces for system configuration and customization (e.g., customization of predefined alarm management policies and patient case sheets for health centers providing specialized services); adopting existing standards (e.g., HL7) for assuring interoperability with hospital back-end systems.

Leveraging on the ERMHAN modular design, future work will also deal in analyzing and integrating existing user interfaces and applications designed according to HCI (Human Computer Interaction) principles, especially interfaces designed for people with cognitive [27] and physical impairments [28].

Such advancements, together with more extensive testing activities, including patients' evaluation of system features, are needed for a final assessment of the proposed platform.

## Figures and Tables

**Table 1:** Alert policy examples.

Alarm level	Alert policies
VERY LOW	(i) SMS to patient relative, no acknowledgement

LOW	(i) SMS and mail to general practitioner, no acknowledgement
	(ii) SMS and mail to relative, no acknowledgement

MEDIUM	(i) SMS and mail to general practitioner or nurse, acknowledgment required
	(ii) Send SMS and mail to relative, no acknowledgment

HIGH	(i) Message to emergency operator, acknowledgment required
	(ii) SMS to patient relative, acknowledgement required

**Table 2:** Mean and standard deviation values of alarm detection (**T**_**ALARM**_), alert policy definition (**T**_**POLICY**_) and management, and overall alarm management times (**T**_**MANAGEMENT**_) in a basic Context Management configuration.

Alarm level	**T**_**ALARM**_		**T**_**POLICY**_		**T**_**MANAGEMENT**_	
	*Time slot 1*	*Time slot 2*	*Time slot 1*	*Time slot 2*	*Time slot 1*	*Time slot 2*
	*Average*		*Average*		*Average*	
Very low	1567 ± 220	1358 ± 213	869 ± 203	843 ± 175	7728 ± 951	6814 ± 1303
	1449 ± 238		854 ± 186		6196 ± 1247	

Low	1457 ± 207	1360 ± 217	879 ± 218	869 ± 203	8235 ± 618	6963 ± 1247
	1409 ± 217		874 ± 210		7604 ± 1168	

Medium	1455 ± 178	1576 ± 220	865 ± 215	869 ± 232	8433 ± 652	7837 ± 680
	1515 ± 208		867 ± 223		8137 ± 728	

High	1402 ± 201	1576 ± 222	859 ± 221	855 ± 225	7481 ± 610	8848 ± 635
	1489 ± 229		857 ± 222		8164 ± 925	
